# Differences in Cardiopulmonary Fitness Between Boy and Girls With Repaired Tetralogy of Fallot

**DOI:** 10.3389/fped.2022.911825

**Published:** 2022-07-06

**Authors:** Yung-Liang Chang, Tzu-Hsuan Kuan, Chia-Hsin Chen, Yi-Ju Tsai, Guan-Bo Chen, Ko-Long Lin, Sheng-Hui Tuan

**Affiliations:** ^1^Department of General Medicine, Kaohsiung Medical University Hospital, Kaohsiung, Taiwan; ^2^Department of General Medicine, E-DA Hospital, Kaohsiung, Taiwan; ^3^Department of Physical Medicine and Rehabilitation, Kaohsiung Medical University Chung-Ho Memorial Hospital, Kaohsiung, Taiwan; ^4^School of Medicine, Kaohsiung Medical University, Kaohsiung, Taiwan; ^5^Institute of Allied Health Sciences, National Cheng Kung University, Tainan City, Taiwan; ^6^Department of Physical Therapy, National Cheng Kung University, Tainan City, Taiwan; ^7^Department of Internal Medicine, Kaohsiung Armed Forces General Hospital, Kaohsiung, Taiwan; ^8^Department of Physical Medicine and Rehabilitation, Kaohsiung Veteran General Hospital, Kaohsiung, Taiwan; ^9^School of Medicine, College of Medicine, National Yang Ming Chiao Tung University, Taipei City, Taiwan; ^10^Department of Rehabilitation Medicine, Cishan Hospital, Ministry of Health and Welfare, Kaohsiung, Taiwan

**Keywords:** tetralogy of fallot, congenital heart disease, cardiopulmonary fitness, gender differences, self-efficacy

## Abstract

**Objective:**

Many studies have demonstrated that patients with repaired tetralogy of Fallot (rTOF) have generally poorer cardiopulmonary fitness (CPF). However, little is known about how the disease results in different CPF in each sex. Therefore, this study aimed to investigate whether sex (and gender) differences affect CPF in children and adolescents with rTOF.

**Methods:**

This retrospective study recruited adolescents and children (aged 10–18) with rTOF following an acute stage of tetralogy of Fallot (TOF) who received cardiopulmonary exercise testing (CPET) in the past 10 years. CPF was determined by symptom-limited CPET with a treadmill under ramped Bruce protocol. Boys and girls were categorized into groups based on body mass index (BMI) and fat mass index (FMI). The BMI was grouped by Taiwanese obesity cutoff points. The FMI was categorized by the body fat percentage. Excess adiposity was defined as (1) “overweight” and “obese” by BMI and (2) greater than the sex- and age-specific 75th percentile of whole subjects by FMI.

**Results:**

A total of 185 participants (104 boys and 81 girls) with rTOF were recruited for the final analysis. Within the BMI category, 76 boys and 63 girls were in the normal group, whereas 28 boys and 18 girls were in the excessive adiposity group. Within the FMI category, 77 boys and 60 girls were in the normal group, whereas 27 boys and 21 girls were in the excessive adiposity group. The analysis of the body composition of rTOF subjects showed that there was no statistically significant difference between the subgroups of the two sexes, but both showed a smaller body size than normal Taiwanese children. When comparing the CPF within different BMI and FMI groups, the children in the excessive adiposity group had significantly lower values in metabolic equivalents (MET) at anaerobic threshold, peak MET, and measured peak oxygen consumption (VO_2_) to predicted peak VO_2_, regardless of sex. Moreover, boys (60.90%) presented poorer CPF than girls (74.22%, *p* < 0.001).

**Conclusion:**

In Taiwan, patients with rTOF had poorer CPF than healthy peers. This study found that girls with rTOF had better CPF than boys with rTOF. The effect of gender stereotypes on sports participation and self-efficacy might be one of the contributing factors; however, further investigations are warranted to determine the causal effect.

## Introduction

Tetralogy of Fallot (TOF), the most common form of cyanotic heart condition, accounts for 7–10% of congenital heart disease (CHD) (1) and affects men and women without significant difference (2). Along with the increasing prevalence of global CHD, the recent prevalence of TOF in Asia, for the first time, appears higher than that in Europe and America. This finding also suggests higher genetic or environmental susceptibility relating to CHD among Asians (3). Depending on disease severity, most surgical repairs of TOF take place between 6 months and one year of age (4). With the improvement of surgery and critical care, the survival rate of treated TOF is excellent (25-year survival following TOF repair: 94.5%) (5). In patients with TOF who underwent total cardiac repair, low early mortality rate and high postoperative survival rate were observed after 30 years (6).

Cardiopulmonary fitness (CPF) is defined as the overall capacity of the cardiovascular and respiratory systems to carry out prolonged strenuous exercise. Cardiopulmonary exercise testing (CPET) involves measurements of respiratory oxygen uptake (VO2), carbon dioxide production (VCO2), and ventilatory measures during a symptom-limited exercise test that permits the evaluation of both submaximal and peak exercise responses (7). Several parameters to indicate CPF could be measured during the CPET. For example, the anaerobic threshold (AT) is an index used to estimate exercise capacity; respiratory exchange ratio (RER), the ratio of VCO_2_/VO_2_, is an index to determine whether the participant achieves maximal effort during the CPET; peak VO_2_, a measure that combines cardiovascular and skeletal muscle oxidative function, is valuable in the objective assessment of exercise tolerance (7). Therefore, CPET is considered the gold standard to measure CPF (8). It can be used to prognostically predict cardiac-related hospitalization and death of patients with repaired TOF (rTOF) as it prevents triggering a potential life-threatening myocardial response in patients with CHD (9–11). Studies have observed compromised CPF among rTOF cohorts (8, 12), and better CPF predicts less cardiac-related hospitalization in 2 years after CPET (13).

Globally, the increasing prevalence of obesity, observed not only in the general population but also in patients with rTOF, shows detrimental effects on cardiac and non-cardiac comorbidities, especially in patients with CHD (14, 15). Although body mass index (BMI) is commonly used to determine obesity, it might be poorly correlated with the true percentage of fat mass (%FM) since it reflects both the fat mass (FM) and fat-free mass (FFM) in the body, especially in children and adolescents (16). Studies have also shown that fat mass index (FMI) better classifies body adiposity compared to BMI (17) and %FM (18). According to a study on Taiwanese subjects, overweight/obese children were found to present with poor CPF from the age at which they begin attending preschool, and differences in CPF between boys and girls were also noted (19). The study also found a negative correlation between CPF and high BMI and FMI; however, the FMI correlated more with peak metabolic equivalent (MET) than the BMI (20).

Therefore, this study aims to investigate differences in the relationship of arthrometry-body composition with CPF in children and adolescents with rTOF based on their sex (and gender).

## Materials and Methods

### Subject Characteristics

This retrospective cohort study was conducted at a tertiary medical center in southern Taiwan. We randomly recruited children and adolescents (aged 10 to 18 years) with TOF who underwent regular follow-up at a pediatric cardiology outpatient clinic between February 2012 and February 2021. The inclusion criteria were children with rTOF who (A) underwent the symptom-limited treadmill exercise test under ramped Bruce protocol after the acute stage of TOF (at least 1 year apart from the latest heart operation), (B) completed transthoracic echocardiographic examination, and (C) underwent standard 12-lead electrocardiogram. The exclusion criteria were (a) age under 10 years old when receiving CPET, (b) recent hospitalization due to heart disease or other major diseases (within 3 months before the date of CPET), (c) incomplete CPET due to muscle fatigue, (d) concurrent known pulmonary disease, and (e) patient's refusal to participate. Basic and clinical data were recorded for all participants, including age, sex, weight, height, BMI, and age at repair. This study was conducted in accordance with the Helsinki Declaration. Ethical approval was obtained from the Institutional Review Board of Kaohsiung Veterans General Hospital, Taiwan (approval number: VGHKS17-CT11-11). The whole study adhered to the STROBE checklist.

### Anthropometry-Body Composition

Zeus 9.9 PLUS (Jawon Medical Co., Ltd., Kyungsang Bukdo, Korea), a body composition analyzer utilizing the tetrapolar electrode method, was used to analyze the body and fat composition through bioelectrical impedance vector analysis. The analyzer calculated the BMI and FMI with the participant's basic data (sex, age, height, weight, and newly calculated body impedance). The participants were then categorized into four groups (underweight, normal, overweight, and obese) according to the age and gender-specific BMI reference values suggested by the Ministry of Health and Welfare in Taiwan (2019) (21, 22). Excess adiposity was defined by FMI >75th percentile of each sex as recommended by Weber et al. (23).

### Cardiopulmonary Exercise Test (CPET)

The MetaLyzer 3B (Cortex Biophysik GmbH Co., Germany) system, which combines a treadmill, a gas analyzer, and an electrocardiographic monitor, was used to measure the patients' CPF and exercise capacity performance. The ramped Bruce protocol was adopted based on the recommendation of the American College of Sports Medicine (ACSM). Throughout the test, the participant's blood pressure, heart rate (HR), respiratory exchange ratio (RER), and min ventilation (VE) were closely monitored. The oxygen consumption (VO_2_) and carbon dioxide production (VCO_2_) were measured using the breath-to-breath method (24). The peak VO_2_ was determined when two of the following three conditions were met: (1) RER > 1.1, (2) HR within 5% of the age-predicted maximum, or (3) the participant is exhausted and refuses to continue despite strong verbal encouragement (25). To measure the peak metabolic equivalents (METs), the peak VO_2_ level was then divided by 3.5 ml/kg/min. Peak VO_2_% was defined as the percentage of the measured peak VO_2_ to the predicted peak VO_2_ after comparing with the normal standards for cardiopulmonary responses to exercise in Taiwan (26). The anaerobic threshold (AT) was derived via the VE/VO_2_ and VE/VCO_2_ methods (27). Informed consent was confirmed and obtained from the parents prior to the test. The test was terminated when participants showed subjective symptoms, expressed their wish to discontinue, or reached the maximal effort as defined by the ACSM (24). All CPET trials were performed under the supervision of an experienced physiatrist (K-LL, who has more than 10 years of experience in CPET) who specialized in CPET.

### Statistical Analysis

Based on the statistical G^*^Power software (version 3.1.9.2 for Windows) and the statistical method used for the study purpose (28), a two-tailed test with 0.8 effect size, alpha of 0.05, and power of 0.80 with equally sized groups yielded a sample size of 52 with 26 participants in each group to detect the effect.

SPSS for Windows version 21.0 (IBM Corp., Armonk, NY, USA) was used for all analyses. Continuous data were presented as mean standard deviation, and categorical variables were presented as absolute numbers or percentages. Before each analysis, normality and homoscedasticity were carefully examined. We first did a 2-way analysis of variance (ANOVA) to test the main effect of sex and body adipose on CPF, and to detect if any interaction between sex and body adipose influenced the parameters of CPF. We did the ANOVA with simple effects via file splitting by sex or body adipose. Since there were only two groups in each variable (sex: boys or girls; body adipose: normal or excessive), if the main effect in 2-way ANOVA was significant, we used an independent *t-test* for normally distributed variables or the Mann–Whitney U test for non-normally distributed variables to compare parameters of CPF between sexes and subjects from different BMI and FMI groups. A *P*-value of < 0.05 was considered statistically significant.

## Results

### Demographic Characteristics

A total of 202 children met the inclusion criteria initially. Among them, seven participants did not have a specific date of the latest heart repair, six participants did not have complete echocardiography data, and four participants had no electrocardiogram data. Eventually, 185 children (104 boys and 81 girls) met the inclusion criteria. Table 1 summarizes the demographic data of all children, presenting a sex-based comparison between the baseline characteristics. We divided our study group according to their body fat, which was quantified by BMI and FMI. As a result, no significant difference was observed between the two groups based on their sex in terms of age (*p* = 0.851), height (*p* = 0.081), weight (*p* = 0.132), and BMI (*p* = 0.780). Boys had a significantly lower fat mass and FMI and higher fat-free mass index than girls (all *p* < 0.05). The BMI of the overall combined overweight and obese children was 24.4% and the excess adiposity FMI was 25.95%.

**Table 1:** Baseline characteristics of patients with repaired tetralogy of fallot aged between 10 and 18 years.

		**Age (year)**	**Height (cm)**	**Weight (kg)**	**BMI (kg/m^**2**^)**	**U (%)**	**N (%)**	**O (%)**	**F (%)**	**FM (kg)**	**FMI (kg/m^**2**^)**	**FFM (kg)**	**FFMI (kg/m^**2**^)**	**Excess adiposity by FMI**
Boy	*N* = 104	12.83 ± 3.89	151.21 ± 20.72	46.20 ± 18.53	19.39 ± 4.09	23.1	50.0	24.0	2.9	8.09 ± 6.72	3.26 ± 2.28	38.10 ± 13.08	16.01 ± 2.19	25.96%
Girl	*N* = 81	12.74 ± 3.62	146.47 ± 16.05	42.67 ± 13.17	19.23 ± 3.65	17.3	61.7	19.8	1.2	10.17 ± 5.08	4.57 ± 1.98	32.49 ± 8.84	14.80 ± 1.94	25.93%
Total	*N* = 185	12.80 ± 3.77	149.14 ± 18.92	44.65 ± 16.45	19.32 ± 3.89	20.5	55.1	22.2	2.2	9.00 ± 6.13	3.83 ± 2.25	35.65 ± 11.73	15.48 ± 2.16	25.95%
*p*-value		0.851	0.081	0.132	0.780	0.418^a^				0.022*	<0.001*	0.001*	<0.001*	0.99*^, *a*^

*BMI, body mass index; U (%), percentage of underweight subjects; N (%), percentage of normal weight subjects; O (%), percentage of overweight subjects; F (%), percentage of fat subjects; FM, fat mass; FMI, fat mass index; FFM, fat-free mass; FFMI, fat-free mass index; Excess adiposity by FMI, >75th percentile of FIM of each sex as per the suggestion of Weber et al. (23).*

a *All the comparisons between girls and boys were done by independent t-test except p-values marked with a, which were analyzed by an independent chi square test for comparison percentage of excess adiposity between girls and boys;*

* *p-value < 0.05*.

### Comparison of CPF Between Boys and Girls With Various BMIs and FMIs

No significant interaction effects of sex and body adipose defined by BMI [F (1,181) = 0.008–1.633, p = 0.203–0.821] in the CPF parameters (AT MET, AT VO_2_, peak MET, peak VO_2_, RER, peak VO_2_%) were found. Similarly, no significant interaction effects of sex and body adipose defined by FMI [F (1,181) = 0.032–2.899, *p* = 0.096–0.858] in the CPF parameters were found either. [F (1,181) = 0.032–2.899, *p* = 0.096–0.858]. The 2-way ANOVA revealed that the main effect of boys was significant [F (1,181) = 6.832–34.499, *p* < 0.001–0.01] with normal body adipose defined by BMI [F (1,181) = 8.973–12.567, *p* < 0.001–0.003] and FMI [F (1,181) = 15.240–23.050, all *p* < 0.001] on parameters of CPF except for RER (Table 2).

**Table 2:** Two-way ANOVA results on the influence of sex, body adipose, and interaction between sex and body adipose on the cardiopulmonary fitness of patients with repaired tetralogy of fallot.

**Parameter**	**Factor**	**Degree of freedom**	**F-value**	***P*-value**
**AT MET (ml/kg/min)**	Sex	1	6.832	0.010*
	BMI	1	12.567	<0.001*
	Sex* BMI	1	1.633	0.203
	Sex	1	2.263	0.134
	FMI	1	23.050	<0.001*
	Sex*FMI	1	0.286	0.593
**ATVO**_**2**_!!!!break!!!! **(ml/min)**	Sex	1	6.832	0.010*
	BMI	1	12.567	<0.001*
	Sex* BMI	1	1.633	0.203
	Sex	1	11.565	0.001
	FMI	1	19.539	<0.001*
	Sex*FMI	1	0.522	0.383
**Peak MET**!!!!break!!!! **(ml/kg/min)**	Sex	1	8.594	0.004*
	BMI	1	8.973	0.003*
	Sex* BMI	1	0.305	0.582
	Sex	1	7.369	0.007*
	FMI	1	19.905	<0.001*
	Sex*FMI	1	1.291	0.257
**Peak VO**_**2**_ **(mi/min)**	Sex	1	10.505	0.001*
	BMI	1	9.294	0.003*
	Sex* BMI	1	1.109	0.294
	Sex	1	16.5.7	<0.001*
	FMI	1	22.735	<0.001*
	Sex*FMI	1	2.899	0.096
**Peak RER**	Sex	1	0	0.999
	BMI	1	1.311	0.254
	Sex* BMI	1	0.008	0.959
	Sex	1	0.319	0.573
	FMI	1	0.692	0.406
	Sex*FMI	1	0.864	0.354
**Peak VO**_**2**_**%**!!!!break!!!! **(%)**	Sex	1	26.946	<0.001*
	BMI	1	10.305	0.002*
	Sex* BMI	1	0.051	0.821
	Sex	1	34.499	<0.001*
	FMI	1	15.240	<0.001*
	Sex*FMI	1	0.032	0.858

*ANOVA, analysis of variance; metabolic equivalent at anaerobic threshold; BMI, body mass index; FMI, fat mass index; Peak MET, peak metabolic equivalent during exercise testing; AT VO2, oxygen consumption at anaerobic threshold; Peak VO2, peak oxygen consumption during exercise testing; RER, respiratory exchange ratio; peak VO2%, percentage of measured peak oxygen consumption to the predicted value.*

* *p-value < 0.05*.

All participants were classified into BMI and FMI subgroups. Each of the groups included patients with normal BMI (76 boys and 63 girls) and excessive BMI (28 boys and 18 girls) and patients with normal FMI (77 boys and 60 girls) and excessive FMI (27 boys and 21 girls). Irrespective of the BMI or FMI grouping, the mean peak RER values exceeded 1.1 (*p* = 0.246 vs. 0.432), suggesting that maximal oxygen exercise efforts were reached. Peak VO_2_% is a relative value described as a percentage compared with normal peers, in which 100% is normal and at least >80% is desired. We observed that the peak VO_2_% of both sexes (mean value: 66.73 ± 15.56, boys and girls: 60.90 ± 12.24 vs. 74.22 ± 16.22) was lower than the desired criterion, indicating that both boys and girls had impaired CPF (Tables 3, 4).

**Table 3:** Comparisons of cardiopulmonary fitness between patients with repaired tetralogy of fallot with normal and excessive body adipose (BMI group).

	**AT MET**	**ATVO_**2**_**	**Peak MET**	**Peak VO_**2**_**	**Peak RER**	**Peak VO_**2**_%**
	**(ml/kg/min)**	**(ml/min)**	**(ml/kg/min)**	**(mi/min)**		**(%)**
Total (*N* = 185)	6.10 ± 1.41	921.39 ± 341.61	8.54 ± 1.93	1,300.36 ± 508.90	1.16 ± 0.10	66.73 ± 15.56
Normal (*N* = 140)	6.23 ± 1.40	866.52 ± 309.25	8.76 ± 1.92	1,228.68 ± 642.49	1.16 ± 0.10	68.85 ± 15.33
Excessive (*N* = 45)	5.71 ± 1.41	1,092.11 ± 382.76	7.85 ± 1.80	1,523.36 ± 583.18	1.14 ± 0.10	60.16 ± 14.57
*P*-value	0.033*	<0.001*	0.005*	0.001*	0.246	0.01*
Boys (*N* = 104)	6.28 ± 1.44	975.18 ± 377.55	8.97 ± 1.93	1,406.91 ± 563.04	1.16 ± 0.10	60.90 ± 12.24
Normal (*N* = 76)	6.43 ± 1.38	901.28 ± 335.65	9.28 ± 1.85	1,313.39 ± 510.43	1.16 ± 0.10	62.84 ± 11.66
Excessive (*N* = 28)	5.84 ± 1.51	1,175.79 ± 416.79	8.14 ± 1.93	1,660.74 ± 627.85	1.14 ± 0.10	55.62 ± 12.42
*P*-value	0.062	0.03*	0.007*	0.005*	0.402	0.007*
Girls (*N* = 81)	5.88 ± 1.37	852.32 ± 276.21	7.99 ± 1.80	1,163.55 ± 392.17	1.16 ± 0.10	74.22 ± 16.22
Normal (*N* = 63)	5.98 ± 1.39	825.24 ± 271.46	8.16 ± 1.85	1,128.08 ± 377.93	1.16 ± 0.10	75.97 ± 16.17
Excessive (*N* = 18)	5.49 ± 1.22	954.28 ± 278.05	7.37 ± 1.49	1,297.09 ± 427.35	1.14 ± 0.11	67.64 ± 15.09
*P*-value	0.192	0.087	0.110	0.115	0.432	0.059

*AT MET, metabolic equivalent at anaerobic threshold; Peak MET, peak metabolic equivalent during exercise testing; AT VO_2_, oxygen consumption at anaerobic threshold; Peak VO_2_, peak oxygen consumption during exercise testing; RER, respiratory exchange ratio; peak VO_2_%, percentage of measured peak oxygen consumption to the predicted value.*

* *p-value < 0.05*.

**Table 4:** Comparisons of cardiopulmonary fitness between patients with repaired tetralogy of fallot with normal and excessive body adipose (FMI group).

	**AT MET**	**ATVO_**2**_**	**Peak MET**	**Peak VO_**2**_**	**Peak RER**	**Peak VO_**2**_%**
	**(ml/kg/min)**	**(ml/min)**	**(ml/kg/min)**	**(mi/min)**		**(%)**
Total (*N* = 185)	6.10 ± 1.41	921.39 ± 341.61	8.54 ± 1.93	1,300.36 ± 508.90	1.16 ± 0.10	66.73 ± 15.56
Normal (*N* = 137)	6.39 ± 1.40	856.25 ± 301.29	8.90 ± 1.92	1,198.67 ± 439.11	1.16 ± 0.10	69.08 ± 15.18
Excessive (*N* = 48)	5.29 ± 1.12	1,107.32 ± 382.77	7.52 ± 1.59	1,590.59 ± 582.92	1.15 ± 0.10	60.04 ± 14.83
*P*-value	<0.001*	<0.001*	<0.001*	<0.001*	<0.001*	<0.001*
Boys (*N* = 104)	6.28 ± 1.44	975.18 ± 377.55	8.97 ± 1.93	1,406.91 ± 563.04	1.16 ± 0.10	60.90 ± 12.24
Normal (*N* = 77)	6.59 ± 1.37	882.32 ± 318.32	9.41 ± 1.83	1,270.13 ± 478.62	1.16 ± 0.10	63.33 ± 11.55
Excessive (*N* = 27)	5.39 ± 1.26	1240.02 ± 412.47	7.73 ± 1.68	1,796.97 ± 610.61	1.14 ± 0.10	53.94 ± 11.64
*P*-value	<0.001*	<0.001*	<0.001*	<0.001*	0.182	<0.001*
Girls (*N* = 81)	5.88 ± 1.37	852.32 ± 276.21	7.99 ± 1.80	1,163.55 ± 392.17	1.16 ± 0.10	74.22 ± 16.22
Normal (*N* = 60)	6.13 ± 1.42	822.79 ± 276.95	8.25 ± 1.84	1,106.96 ± 366.27	1.16 ± 0.10	76.45 ± 16.15
Excessive (*N* = 21)	5.17 ± 0.90	936.71 ± 262.29	7.25 ± 1.44	1,325.24 ± 427.06	1.16 ± 0.10	67.87 ± 15.03
*P*-value	0.005*	0.104	0.028*	0.027*	0.949	0.036*

*AT MET, metabolic equivalent at anaerobic threshold; Peak MET, peak metabolic equivalent during exercise testing; AT VO_2_, oxygen consumption at anaerobic threshold; Peak VO_2_, peak oxygen consumption during exercise testing; RER, respiratory exchange ratio; peak VO_2_%, percentage of measured peak oxygen consumption to the predicted value.*

* *p-value < 0.05. Normal group <75% percentile of FIM of the each tested sample of sex; Excessive group≥75% percentile of FIM of the each tested sample of sex*.

Table 3 provides a comparison of the CPF of patients categorized based on their BMI. Traditionally, patients with excessive BMI might have greater measured oxygen consumption at AT VO_2_ and peak VO_2_ value, indicating that a greater body weight follows a greater oxygen consumption. In the boys' subgroup, we observed that AT VO_2_ and peak VO_2_ values followed the traditional rule (both *p* < 0.05); however, in the girls' subgroup, no statistically significant differences in AT VO_2_ and peak VO_2_ values were found between patients with normal and excessive BMI (both *p* > 0.05). Moreover, boys with normal BMI had significantly higher peak MET (*p* = 0.005) and relatively higher AT MET (*p* = 0.062) than those with excessive adiposity. Surprisingly, in the girls' subgroup, the values of AT MET (*p* = 0.192) and peak MET (*p* = 0.115) did not show statistically significant differences.

Table 4 presents a comparison of the AT VO_2_ and peak VO_2_ values in patients categorized based on their FMI. The AT VO_2_ and peak VO_2_ value in the boys' subgroup showed statistically significant differences between patients with normal adiposity and excessive adiposity (both *p* < 0.001). The girls' subgroup showed relatively higher AT VO_2_ (*p* = 0.083) and peak VO_2_ (*p* = 0.060) values in patients with excessive adiposity than those with normal adiposity. As expected, both boys and girls with normal adiposity had higher AT MET and peak MET than those with excessive adiposity (all *p* < 0.05).

### Comparison of CPF Between Sexes With Normal and Excessive Body Adipose

Table 5 provides a comparison of the CPF between girls and boys with various BMIs and FMIs. Boys had a higher AT VO_2_, peak MET, and peak VO_2_ than girls (p-values from 0.001 to 0.015) but comparable AT MET. As for the normal BMI and FMI subgroups, boys had higher peak MET and peak VO_2_ than girls (*p*-values from < 0.001 to 0.030) but comparable AT VO_2_ and AT MET. With regard to the excessive BMI and FMI groups, boys had higher AT VO_2_ and peak VO_2_ than girls (*p*-values from 0.004 to 0.041) but comparable AT MET and peak MET. As for the peak VO_2_%, girls with rTOF presented a higher significant peak VO_2_% than boys with rTOF regardless of different BMI or FMI subgroups (*p*-values from < 0.001 to 0.006).

**Table 5:** Comparisons of cardiopulmonary fitness between sexes with normal and excessive body adipose among patients with repaired tetralogy of fallot.

**Total (Normal + Excess)**	**AT MET (ml/kg/min)**	**ATVO_**2**_** **(ml/min)**	**Peak MET (ml/kg/min)**	**Peak VO_**2**_** **(mi/min)**	**Peak RER**	**Peak VO_**2**_%** **(%)**
Boys (*N* = 104)	6.28 ± 1.44	975.18 ± 377.55	8.97 ± 1.93	1,406.91 ± 563.04	1.16 ± 0.10	60.90 ± 12.24
Girls (*N* = 81)	5.88 ± 1.37	852.32 ± 276.21	7.99 ± 1.80	1,163.55 ± 392.17	1.16 ± 0.10	74.22 ± 16.22
*P*-value	0.060	0.015*	0.001*	0.001*	0.893	<0.001*
**Normal** ***BMI***	**AT MET (ml/kg/min)**	**ATVO**_**2**_ **(ml/min)**	**Peak MET (ml/kg/min)**	**Peak VO**_**2**_ **(mi/min)**	**Peak RER**	**Peak VO**_**2**_**%** **(%)**
Boys (*N* = 76)	6.43 ± 1.38	901.28 ± 335.65	9.28 ± 1.85	1,313.39 ± 510.43	1.16 ± 0.10	62.84 ± 11.66
Girls *(N* = 64)	5.98 ± 1.39	825.24 ± 271.46	8.16 ± 1.85	1,128.08 ± 377.93	1.16 ± 0.10	75.97 ± 16.17
*P*-value	0.057	0.148	<0.001*	0.018*	0.927	<0.001*
**Normal** ***FMI***	**AT MET (ml/kg/min)**	**ATVO**_**2**_ **(ml/min)**	**Peak MET (ml/kg/min)**	**Peak VO**_**2**_ **(mi/min)**	**Peak RER**	**Peak VO**_**2**_**%** **(%)**
Boys (*N* = 77)	6.59 ± 1.37	882.32 ± 318.32	9.41 ± 1.83	1,270.13 ± 478.62	1.16 ± 0.10	63.34 ± 11.55
Girls (*N* = 60)	6.13 ± 1.42	822.79 ± 276.95	8.25 ± 1.85	1,106.96 ± 366.27	1.16 ± 0.10	76.45 ± 16.15
*P*-value	0.058	0.253	<0.001*	0.030*	0.721	<0.001*
**Excess** ***BMI***	**AT MET (ml/kg/min)**	**ATVO**_**2**_ **(ml/min)**	**Peak MET (ml/kg/min)**	**Peak VO**_**2**_ **(mi/min)**	**Peak RER**	**Peak VO**_**2**_**%** **(%)**
Boys (*N* = 28)	5.84 ± 1.52	1,175.79 ± 416.79	8.14 ± 1.93	1,660.74 ± 627.85	1.14 ± 0.10	55.62 ± 12.42
Girls (*N* = 17)	5.49 ± 1.22	954.28 ± 278.05	7.37 ± 1.49	1,297.09 ± 427.35	1.14 ± 0.11	67.64 ± 15.08
*P*-value	0.427	0.038*	0.169	0.041*	0.959	0.006*
**Excess** ***FMI***	**AT MET (ml/kg/min)**	**ATVO**_**2**_ **(ml/min)**	**Peak MET (ml/kg/min)**	**Peak VO**_**2**_ **(mi/min)**	**Peak RER**	**Peak VO**_**2**_**%** **(%)**
Boys (*N* = 27)	5.39 ± 1.26	1,240.02 ± 412.47	7.73 ± 1.68	1,796.97 ± 610.61	1.14 ± 0.10	53.94 ± 11.64
Girls (*N* = 21)	5.17 ± 0.91	936.71 ± 262.29	7.25 ± 1.44	1.325.24 ± 427.06	1.16 ± 0.10	67.87 ± 15.03
*P*-value	0.509	0.003*	0.309	0.004*	0.389	0.001*

*AT MET, metabolic equivalent at anaerobic threshold; Peak MET, peak metabolic equivalent during exercise testing; AT VO_2_, oxygen consumption at anaerobic threshold; Peak VO_2_, peak oxygen consumption during exercise testing; RER, respiratory exchange ratio; peak VO_2_%, percentage of measured peak oxygen consumption to the predicted value, BMI, body mass index; FMI, fat mass index; Excess BMI, overweight and obesity BMI; Excess FMI, >75th percentile of FIM of each sex as per the suggestion of Weber et al. (23).*

* *p-value < 0.05*.

## Discussion

While offering some novel insights, the findings of this study are consistent with some existing research (8, 12, 29). Although both boys and girls with rTOF who had undergone cardiac surgery presented with reduced CPF in a previous study (29), we found that girls with rTOF had better CPF than boys with rTOF. Apart from surgical selection and developing techniques, we believe that other factors like sports participation and self-efficacy impact CPF. For example, the effect of gender stereotype and caregiver stress might be a determinant for boys with rTOF having poorer CPF than girls with rTOF. However, in this retrospective study, we could not investigate some factors, including the onset age of TOF, TOF severity, age of the participant during the total repair surgery for TOF, surgeon's technique, postoperative condition of the patient, follow-up of cardiac function by cardiac echo, the onset of puberty, or the current status of puberty level of our subjects. Moreover, we could not determine which factor contributed more to the differences in CPF in our patients.

We analyzed the relationship between BMI, FMI, and CPF in Taiwanese children and adolescents with rTOF aged between 10 and 18 years based on their sex. According to a previous study, the prevalence of childhood obesity is generally higher in southern Taiwan than in other regions of Taiwan (30). Our results showed that the percentage of overweight and obese children (boys, 26.9%; girls, 21.1%) was slightly less than that of the national survey in Taiwan (boys, 30.5%; girls, 22.2%) (21, 22). Some studies have indicated that patients with rTOF might have preoperative malnutrition problems and postoperative long-term growth restrictions (31–33). It is reasonable to observe that the percentage of overweight and obesity in our participants was relatively lower than their healthy peers. To better describe obesity in pubertal patients, we also investigated our participants' FMI for a more accurate comparison, as suggested by a previous study (17). In our study, the prevalence of excess body weight defined by FMI (boys, 25.96%; girls, 25.93%) was similar to the prevalence of overweight and obesity defined by BMI (boys, 26.9%; girls, 21.1%). However, we could not perform an accurate analysis of the participants' body size through FMI due to the lack of nationwide FMI data in Taiwan. Therefore, further larger and nationwide studies providing reference to FMI values among Taiwanese children and adolescents are necessary for a more precise comparison.

Previous studies in different countries demonstrated that obese (defined by BMI or FMI) children or adolescents have lower CPF than those with normal body weight (19, 34, 35). Researchers even found a strong negative association between CPF levels and BMI in Chinese and Taiwanese children (36, 37). In our study, we found that in each subgroup of boys and girls with rTOF, obese children had poorer CPF than those with normal body weight. Girls with obesity defined by BMI did not have a statistically significantly poorer CPF than those with normal BMIs. However, the resulting CPF value corresponded with the conclusion obtained in previous studies when we defined obesity through excessive adiposity by FMI (19, 34, 35). To explain this phenomenon, previous studies indicated that FMI tends to play a better role in describing obesity than BMI among pubertal children. Both boys and girls would undergo body fat changes before and after puberty (38, 39). A systematic review and meta-analysis also found that BMI might have low sensitivity and failed to differentiate over 25% of children (aged 4–18 years old) with excessive adiposity (40). Therefore, our results are predictable and correspond with a previous study showing that FMI is better than BMI at identifying adiposity in pubertal children (17). Surprisingly, we observed that girls with rTOF had better CPF than boys. This result could not be attributed to body composition differences between boys and girls with rTOF as no statistically significant differences were observed when comparing weight, height, BMI, BMI category, and excessive adiposity by FMI. Although boys and girls predictably showed impaired CPF (29), it was an accidental consequence that boys showed poorer CPF. Hence, there should be factors in our investigation that led to this result.

Self-efficacy is believed to be reduced in patients experiencing severe diseases, such as acute lymphoblastic leukemia. Previous nationwide studies using questionnaires or interviews discussed how an illness would affect a child and the people that surround him/her (41). These experiences were found to play a big role in affecting self-efficacy toward physical activity (24). Some studies have also reported that patients with heart diseases might be exposed to particular surroundings or environmental factors that greatly influence self-efficacy toward physical activity, such as suggestions from cardiologists, expectations from society, and anxiety of the main caregivers (42–44). In our study, the patients underwent cardiac surgery in their childhood, so it is reasonable for them to have limited physical activity as recommended by their cardiologists. The parents are also more aware of the intensity of physical activity the patient is permitted immediately following cardiac surgery. Furthermore, with regard to the differences in self-efficacy between boys and girls with CHD, the cultural stereotyping of sports participation regarding physical strength and prowess, body image, and opportunities for team support has been found to limit the choices for boys and may therefore contribute to their lower sports-related self-efficacy than girls with CHD. This means that girls with rTOF might show a more vigorous resistance than boys (42, 45, 46). While no significant association is reported between disease type or severity and physical activity or self-efficacy in patients with CHD, sociocultural factors are likely to play a predominant role in children and adolescents with rTOF (47, 48).

This study has certain limitations. Our research was conducted in a single medical center in southern Taiwan; thus, the results might be generalized to specific populations. Furthermore, patients with relatively severely impaired cardiac function who could not endure CPET were excluded from this study. However, the population and composition of patients with rTOF might be affected and even influence the results. We did not record the data from a cardiac image, such as echocardiography and cardiac magnetic resonance imaging. Therefore, we are not sure if there is any difference in the prognostic cardiac image indicators between boys and girls. In addition, this study did not qualitatively analyze physical activity or self-efficacy. Finally, there are insufficient nationwide cross-sectional surveys concerning gender differences in sociocultural issues or gender stereotypes in Taiwan. Future prospective cohort studies should conduct regular CPET after the surgery to evaluate the continuous changes in CPF. In addition, cross-sectional surveys through questionnaires or interviews are needed to determine the physical condition and sociocultural expectations of Taiwanese children and adolescents with rTOF.

## Conclusion

Girls with rTOF had a better peak VO_2_% value than boys, suggesting that they have better CPF. No significant difference was found between the BMI categories and excessive adipose tissue by FMI in each sex. Surprisingly, obesity was excluded from the factors leading to various CPF between boys and girls. Therefore, the association between rTOF and self-efficacy in addition to the physical condition and sociocultural expectations may contribute to better CPF in girls with rTOF. More studies are needed to further evaluate the correlations of the above factors.

## Data Availability Statement

The raw data supporting the conclusions of this article will be made available by the authors, without undue reservation.

## Ethics Statement

Ethical approval of this study was obtained from the Institutional Review Board of Kaohsiung Veterans General Hospital, Taiwan (approval number: VGHKS17-CT11-11). Written informed consent to participate in this study was provided by the participants' legal guardian/next of kin.

## Author Contributions

Conceptualization: Y-LC, K-LL, and S-HT. Data curation: T-HK, Y-LC, and S-HT. Methodology: C-HC, Y-JT, and G-BC. Resources: Y-JT and K-LL. Supervision: C-HC and K-LL. Writing—original draft: Y-LC and T-HK. Writing—review and editing: G-BC and S-HT. All authors contributed to the article and approved the submitted version.

## Conflict of Interest

The authors declare that the research was conducted in the absence of any commercial or financial relationships that could be construed as a potential conflict of interest.

## Publisher's Note

All claims expressed in this article are solely those of the authors and do not necessarily represent those of their affiliated organizations, or those of the publisher, the editors and the reviewers. Any product that may be evaluated in this article, or claim that may be made by its manufacturer, is not guaranteed or endorsed by the publisher.

## Abbreviations

CHD: congenital heart disease
TOF: Tetralogy of Fallot
rTOF: repaired Tetralogy of Fallot
CPET: cardiopulmonary exercise testing
CPF: cardiopulmonary fitness
BMI: body mass index
FMI: fat mass index
HR: heart rate
RER: respiratory exchange ratio
VE: min ventilation
VO_2_: oxygen consumption
VCO_2_: carbon dioxide production
MET: metabolic equivalent
AT: anaerobic threshold
ACSM: American College of Sports Medicine
Peak VO_2_%: were defined as the percentage of the measured peak VO_2_ to the predicted peak VO_2_
AT VO_2_: measured oxygen consumption at anaerobic threshold.
